# The impact of maternal flavivirus infections on fetal neurological outcomes: a scoping review

**DOI:** 10.1186/s12985-025-03004-1

**Published:** 2026-01-30

**Authors:** Ved Shanbhag, Zing Len, Shivi Kumar

**Affiliations:** 1 Virginia Polytechnic Institute and State University, Blacksburg, Virginia United States; 2 Midland College, Midland, Texas United States; 3 Marcus High School, Flower Mound, Texas, United States

**Keywords:** Congenital zika syndrome, Diagnostic challenges, Flavivirus, Fetal brain development, Maternal infection

## Abstract

This scoping review examines the neurological effects of maternal flavivirus infection, specifically Zika virus (ZIKV), Dengue virus (DENV), and Yellow Fever virus (YFV), on fetal brain development. Inclusion criteria focused on studies involving maternal infection and neurodevelopmental outcomes in offspring up to five years of age. The review found that ZIKV, especially the African Strain, exhibited neuroviolence and pronounced fetal brain abnormalities. Despite the presence of transplacental maternal transfer, these antibodies did not fully prevent congenital malformations, including microcephaly and developmental delays. Diagnostic limitations, such as serological cross-reactivity, particularly in regions where multiple flaviviruses co-circulate, were shown to impede effective clinical management. Additionally, the review highlights stark disparities in health infrastructure and prenatal care in Latin America, where the burden of Congenital Zika Syndrome (CZS) is disproportionately high. Findings emphasize the need for strain-specific diagnostics and therapeutics, along with long-term cohort studies that integrate virological, immunological, and socio-environmental perspectives. Ultimately, this review underscores the urgent need for equitable public health strategies and continued interdisciplinary research to address the teratogenic risks associated with maternal flavivirus infections.

## Introduction

Flaviviruses represent a significant global health concern due to their potential for widespread transmission and severe clinical outcomes [1]. Among them, Zika virus (ZIKV), Dengue virus (DENV), and Yellow Fever virus (YFV) have garnered attention for their impact on maternal and fetal health [2]. The emergence of ZIKV in the Americas, particularly in Latin America, has been linked to an increase in congenital neurological disorders, including microcephaly, autism spectrum disorders, and cognitive impairments [3]. The pathophysiology of flavivirus infections in pregnant women remains an evolving field, with research efforts focused on understanding transplacental viral transmission, maternal immunological responses, and the subsequent neurodevelopmental impact on the fetus [4]. This systematic review aims to synthesize the existing literature on flavivirus infections in pregnancy, critically evaluate their effects on fetal brain development, and assess the methodologies employed in these studies. Flaviviruses are a genus of RNA viruses primarily transmitted by arthropods, such as Aedes aegypti and Aedes albopictus mosquitoes, which are endemic to tropical and subtropical regions, particularly Latin America [5]. The emergence of ZIKV in 2015–2016 raised urgent concerns about its teratogenic effects, most notably.

Congenital Zika Syndrome (CZS), characterized by microcephaly, neurodevelopmental delays, and structural brain abnormalities [6]. While ZIKV has been directly implicated in fetal neurological impairment, the potential role of other flaviviruses, such as DENV and YFV, remains less understood [7]. Co-circulation of these viruses poses diagnostic challenges due to serological cross-reactivity and may contribute to immune-mediated neurological sequelae [8]. Despite the significant public health burden associated with flavivirus infections in pregnancy, gaps remain in understanding the precise mechanisms of viral transmission and fetal neurodevelopmental disruption [9]. The variation in clinical outcomes based on the timing of maternal infection, viral strain, and host immune response necessitates a comprehensive analysis of existing studies [10]. Additionally, while surveillance efforts have improved, epidemiological data on flavivirus-related fetal outcomes, particularly in Latin American regions, remain inconsistent [11]. The objective of this scoping review is to synthesize and analyze the available literature to identify gaps in different sectors of maternal flavivirus infections, such as resource availability, diagnostic testing, and healthcare literacy, among other factors, to understand better how flavivirus infections can lead to neurological conditions, as well as to evaluate the effects of maternal flavivirus prevalence in endemic regions such as Latin America [12]. This scoping review aims to address these gaps by describing the impact of flavivirus infections in pregnant women, assessing the neurological outcomes in exposed neonates, and identifying the immunological and virological factors that influence disease severity.

## Materials and methods

### Eligibility criteria

This scoping review followed the Preferred Reporting Items for Systematic Reviews and Meta-Analyses extension for Scoping Reviews (PRISMA—ScR) Checklist by Tricco AC et al. The checklist was created as a draft paper and used to aid in organizing a review protocol. It adhered to the Arksey and O & # 39; Malley framework [13], which was further supported through the methodological recommendations outlined by Levac et al. [14].

The inclusion criteria of this study consist of an eligibility criterion. For articles to be included in this review, they needed to be published or accessible in English, and papers were required to focus on specific dimensions of flavivirus infections in pregnant women, including but not limited to Zika virus, dengue virus, and West Nile virus. Papers were included if they compared outcomes of offspring in infected mothers, with a focus on neurodevelopment in children from birth to around 5 years, longitudinal study.

Journals were included if they were conducted as primary studies (e.g., cohort studies, case-control studies, cross-sectional analyses). Papers that focus on non-pregnant populations or unrelated viral infections were excluded, as well as those that examine outcomes of neurodevelopment beyond parameters set for this scoping review, as it would require further qualitative analysis and screening to avoid colluding with the methods and information for this scoping review.

### Information sources

To identify potentially irrelevant documents, a series of three databases was drafted and reviewed by three authors, Shivi Kumar, Ved Shanbhag, and Zing Len, with advanced filters applied, which include texts published in a 10-year interval from December 2014 to December 2024, and texts available with non-charge access (free-full text). Search strategies included research equations referenced in the introduction, and the final search results were exported into Zotero. The three authors extracted information, and any conflicts were resolved by the mitigation of a fourth independent reviewer, Lisbeth Tolentino. Duplicates were removed, and the remaining articles were drafted using a Google spreadsheet.

### Search

The following table presents the research strategy used in PubMed:

### Search strategy used

("flavivirus infection" OR "Zika virus" OR "Dengue virus" OR "Yellow Fever virus") AND ("pregnant women" OR "maternal infection") AND ("fetal brain development" OR "microcephaly" OR "cognitive impairment")

The application of specific keywords incorporated into Boolean operators across three different sources allowed us to search gray literature (Google Scholar) and index articles (PubMed and ScienceDirect). Each author peer-reviewed the research strategy across all sources to ensure that all databases were being processed identically, including the filters applied to all sources that ensured the information is comprehensive and effective to date.

### Selection of sources of evidence

Articles extracted on a Google spreadsheet were alphabetically organized and split into groups of three, with each author reviewing 40 articles. Each author manually screened papers and extracted data, and results and amendments to screenings were made. Results and all screening processes were done in a shared file, allowing for consistency in the discussion of results among authors.

### Data charting process

Data Extraction was done independently by three authors following discussions of results in three total screenings. Charting was done in separate Google Sheets.

### Data extraction

**Fig. 1:**
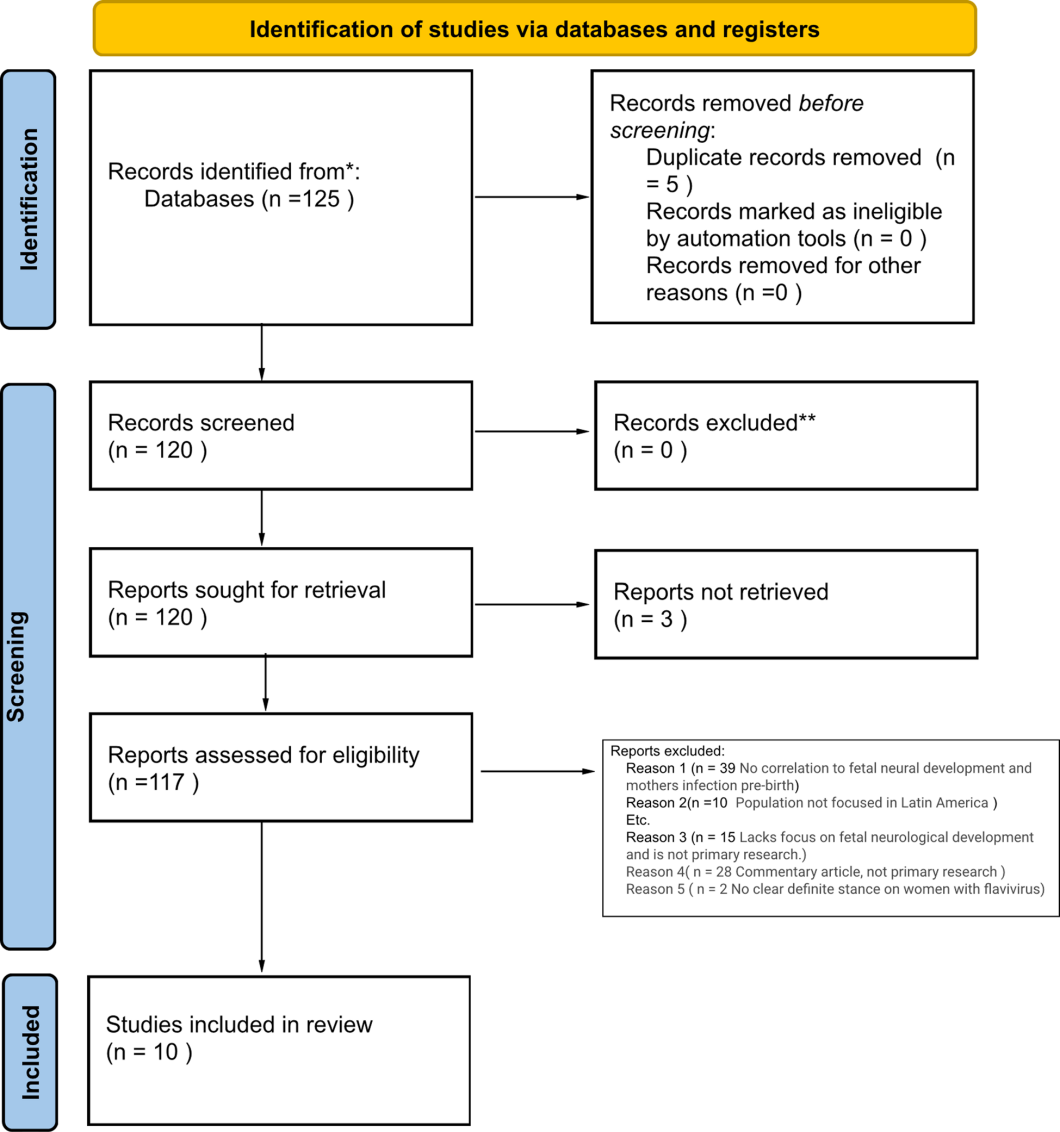
PRISMA Flow Diagram of Study Selection Process This figure illustrates the PRISMA (Preferred Reporting Items for Systematic Reviews and Meta-Analyses) flowchart outlining the selection process for studies included in the scoping review. A total of 125 records were identified from databases, with 5 duplicates removed. After screening 120 records, 3 could not be retrieved, and 117 were assessed for eligibility. Of these, 107 were excluded for reasons such as lack of relevance to fetal neurodevelopment, non-primary research, or inappropriate population focus. Ultimately, 10 studies met the inclusion criteria and were incorporated into the final review

Abstractions of data focused on article characteristics such as year and author, as well as contextual factors such as socioeconomic levels in population-focused as well as the demographic the study focused on. Factors of influence in the conduction of the study, including study design, as well as topics diagnostic testing, prevention, and other factors of flavivirus infections in pregnant women assessment in articles were assessed in analysis to the results concluded by the article (e.g., consequences, implications, and benefits) Fig 1.

Consider, if feasible to do so, reporting the number of records identified from each database or register searched (rather than the total number across all databases/registers).

**If automation tools were used, indicate how many records were excluded by a human and how many were excluded by automation tools.

## Results- demographic and summary

### Demographics

Seven studies were eligible for inclusion. Before 2020, there were three articles on maternal flavivirus infection and transmission mechanisms addressing fetal neurological development. Additionally, there were only four articles from 2020 to 2024, indicating stagnant discussion and research on flavivirus infections and their effect on maternal and fetal transmission. Based on the population published in the articles, the results can only be applied to regions of Latin America, mainly in concentrated outbreak regions. With data availability extracted from ” Bio-surveillance in areas with high infectious potential and bacteriological surveillance study of insects [15] can help to identify mosquito vectors and reservoirs” [16].

### Summary

Based on the similarities, three studies relied heavily on experimental animal models as evidence of linkage between flavivirus and maternal and fetal transmission and pathogenisis” [16–19]. While two articles discussed the difference in symptoms of infection between neonates and adults [17, 20], with one article focusing on the modality and biotechnology available to neutralize and prevent viral infection [21].

The primary mechanism identified across all seven articles was maternal-to-child transmission (MCTC) through the placental and blood-brain barrier (Hcini, 2020). All seven articles address different modes of transmission. Limited preoperative interventions have been discussed in clinical studies. Research in clinical studies focused on the diagnostics (screening, monitoring), epidemiology, and outcomes of flavivirus infection on fetal neurological development rather than interventions.

There is also a common proposal of a direct link between maternal flavivirus infection and congenital abnormalities based on evidence of studies in pre- and postnatal cases, which range from olfactory sensory loss, microcephaly, and even fetal demise [17, 20].

Of which, in Schrauf et al. article Current Efforts in the’ Development of Vaccines for the Prevention of Zika and Chikungunya Virus Infections,” there is an indication that the pro-inflammatory response is a leading cause of the vertical transmission of neurological disease and development. Most commonly discussed neurological development of microcephaly due to cytokinesis [20], and other neurological abnormalities such as Schizipreneina [17].

Additionally, pathogenesis in all seven articles addresses cytokinesis [20] and neural progenitor cells [17], leading to neurogenesis impairment—clinical evidence in tissue tropism [16, 20].

## Discussion

### Comparative severity of African and Asian ZIKV strains

In this scoping review, seven primary study articles address the effect of maternal flavivirus infection on fetal neurological development. Our findings indicate that African and Asian ZIKV strains are actually capable of crossing thebarrier and infecting fetal tissues. However, as per our initial hypothesis, The African strain demonstrated higher pathogenicity, as it exhibited increased viral.

placental titers in fetal tissues, increased inflammation in placental tissues, increased intrauterine (inter-sibling) infectiousness, as well as significant fetal brain weight reduction that indicates a stronger neurotropic effect. These observations are in consonance with previous animal model research, such as Narasimhan et al.[18], where the lineage of the ZIKV strain was proven to be a determining factor in the establishment of fetal severity of disease. The increased placental immune response seen with the African strain also corresponds with findings presented by Elgueta et al.[17] on maternal immune activation and increased fetal neuroinflammation, and disruption of development.

### Fetal neurodevelopment and congenital Zika syndrome (CZS)

Our hypothesis assumed that maternal IgG transfer might in some way protect the fetus, but clinical evidence had some other things to say. Data from human pregnancy cohorts, for example, that of Hcini et al.[16], showed that indeed maternal antibodies were transferred transplacentally but were not necessarily able to prevent congenital malformations like microcephaly, seizure, or neurodevelopmental delay. These observations were substantiated by Wang et al. [19], who offered structural and cellular evidence of ZIKV-induced microcephaly through impaired neural progenitor function. This difference between predicted immunity and discovered teratogenicity underlines the complexity in the interplay between maternal immunity and viral pathogenicity. Our results also validate Afolabi et al.[21], who concluded that maternal infection at first and second trimesters is significantly associated with negative fetal outcomes.

### Public health and epidemiological considerations

Surveillance studies review highlighted the ongoing diagnostic issues in ZIKV-endemic regions. Serological cross-reactivity with flaviviruses of related species, including dengue and chikungunya, remains a difficult obstacle. Improved molecular diagnostics and region-specific detection strategies are urgently needed, as reported by Afolabi et al.[21], and further highlighted by Schrauf et al.[20]. The unequal risk of CZS among socioeconomically disadvantaged populations was also emphasized in both Hcini et al.[16] and Tricco et al. [22], where limited prenatal care, vector control, and health infrastructure exacerbate disease outcomes. These disparities call for public health equity in the implementation of ZIKV prevention and care interventions.

### Implications for future research and public health policy

The established differences in strain virulence also highlight the importance of creating lineage-specific therapeutics and vaccines. Schrauf et al.[20] described the challenge of coming up with vaccines with the best long-term immunity while maintaining safety and adequate immunogenic response. Our findings are in line with this trend, particularly with strain-specific neurovirulence affecting fetal risk profiles. Socio-economic susceptibility should also be integrated into policy formulation and fund prioritization to achieve tailored interventions in susceptible, under-resourced populations. As Elgueta et al.[17] and Chitu et al.[23] indicate, the intersection of virology, immunology, and developmental neuroscience will have to remain central to future research agendas addressing arboviral infection in pregnancy.

### Limitations

Although this scoping review has access to both experimental and clinical data, several limitations must be kept in mind. A lot of the insight comes from animal models, which may not necessarily capture the complexity of human maternal-fetal interactions. Furthermore, heterogeneity in study design, diagnostic criteria, and follow-up length prevents direct cohort comparisons. Long-term follow-up of human neurodevelopment is currently lacking, particularly in low-resource settings where CZS outcomes are likely underreported. Future studies must fill these gaps with standardized protocols, larger sample sizes, the inclusion of socioeconomic factors, and the protective functions and vulnerability of modes of transmission, such as the vulnerability of the placenta, in order to mitigate abnormalities in neurological outcomes.

## Conclusion

This study points out the differential pathogenicity of African and Asian ZIKV strains during pregnancy, where the African strain was more neurovirulent, presented higher fetal viral loads, and had higher placental inflammation in experimental models. These findings endorse the proposal that viral lineage critically determines fetal outcomes and emphasize the potential of strain-specific teratogenicity.

Clinical experience, particularly observations of human pregnancy, confirms the laboratory findings by presenting uniform risks of congenital malformation, in spite of maternal IgG antibody transfer. Complex immunological interactions at the maternal-fetal interface and uniform diagnostic difficulties in endemic regions present serious challenges in the treatment and prevention of Congenital Zika Syndrome (CZS).

The public health consequences are serious, particularly in resource-constrained environments where the burden of disease is out of proportion. This underscores the importance of high-priority focused surveillance, improved diagnostic capabilities, and equitable vaccine development strategies with consideration of viral heterogeneity and socio-economic risk.

Future research must continue to integrate animal and human studies, enhance long-term developmental follow-up of infected infants, and prioritize interdisciplinary approaches to understanding and preventing the full spectrum of ZIKV-related pregnancy complications.

## Data Availability

All the data in the figures is created by us. All the other relevant data can be found using our bibliography section.
